# Phase-II-Studie zur Therapiedeeskalation bei HPV-assoziierten Oropharynxkarzinomen. Kommentar II

**DOI:** 10.1007/s00066-022-01948-9

**Published:** 2022-05-13

**Authors:** Robert Michael Hermann, Hans Christiansen

**Affiliations:** 1 Zentrum für Strahlentherapie und Radioonkologie, Bremen/Westerstede, Deutschland; 2 Klinik für Strahlentherapie und Spezielle Onkologie, OE 8240, Medizinische Hochschule Hannover, Carl-Neuberg-Str. 1, 30625 Hannover, Deutschland

## Hintergrund und Zielsetzung

HPV-assoziierte (also p16-positive) Oropharynxkarzinome haben eine signifikant bessere Prognose als p16-negative Karzinome, sodass die TNM-Klassifikation diese Tumoren seit 2017 in beide Entitäten unterteilt und distinkt voneinander unterschiedlichen Prognosegruppen zuweist. Die günstige Prognose der p16-positiven Tumoren wurde in diversen Studien auch auf ein schnelles und ausgeprägtes Ansprechen auf eine Radio(chemo)therapie (RCT) zurückgeführt. Insofern ist es fraglich, ob bei diesen Tumoren die etablierten Konzepte einer voll dosierten RCT und damit auch die entsprechend assoziierten Toxizitäten wirklich notwendig und ob Deeskalationen dieser Therapieansätze möglich sind. In der hier kommentierten Phase-II-Studie (E3311; NCT01898494) wurde ein pragmatischer Ansatz zur Deeskalation der adjuvanten Therapie bei p16-positiven cM0-Oropharynxkarzinomen nach transoraler Chirurgie getestet. Dabei wurde die adjuvante Therapie nach postoperativ determinierten Risikogruppen adaptiert appliziert. Alexander Rühle und Nils H. Nicolay sind im Februarheft von *Strahlenther Onkol* (Vol. 198 [2022], S. 400–403) bereits dieser wichtigen Frage nachgegangen. Eine kritische Zweitmeinung erscheint dem Herausgeber der Literaturkommentare in unserer Zeitschrift angemessen.

## Studiendesign

Patienten mit Oropharynxkarzinomen im Stadium III–IVA wurden zunächst transoral möglichst vollständig reseziert. Anschließend wurden sie in Risikogruppen unterteilt: Als „niedriges Risiko“ wurde dabei ein pT1–2-Stadium mit negativen Resektionsrändern (mindestens 3 mm) und pN0 oder pN1 (1 LK-Metastase bis maximal 3 cm, ohne extranodales Wachstum [ECE], kein perineurales Wachstum [PNI] oder lymphovaskuläre Infiltration [LVI]) definiert. Diese Patienten wurden nur nachgesorgt, sie erhielten keine adjuvante Therapie (Arm A).

Ein „hohes Risiko“ bedeutete – unabhängig vom T‑Stadium – mindestens einen positiven Schnittrand oder „extensive ECE“ (> 1 mm) oder mehr als 4 befallene Lymphknoten. Diese Patienten erhielten eine adjuvante RCT (normofraktioniert 66 Gy, 7 Zyklen wöchentlich Cisplatin 40 mg/m^2^ – Arm D). Ein „mittleres Risiko“ wurde unterstellt bei einem der folgenden Befunde: knappe Resektionsränder (< 3 mm), minimale ECE (< 1 mm), mindestens 1 LK-Metastase > 3 cm, 2–4 LK-Metastasen (< 6 cm) oder PNI/LVI. Diese Patienten wurden randomisiert zwischen einer „Standardtherapie“ (normofraktioniert 60 Gy – Arm C) und einer „deeskalierten Therapie“ (50 Gy – Arm B). Dabei wurde auch nach Raucherstatus stratifiziert.

Die Studie wurde auf „Nichtunterlegenheit“ des Arms B zu Arm C in Bezug auf das 2‑Jahres-progressionsfreie Überleben (PFS) geplant. Neben diesem onkologischen Endpunkt wurden auch die Lebensqualität und die Schluckfähigkeit erfasst.

Für die Definition des Tumorbett-Boosts war die intraoperative invasive Tumorbasis entscheidend (nicht die präoperative radiologische intraluminale Tumorausbreitung), erweitert um einen Sicherheitsabstand von 3 bis 5 mm.

Die Definition der zervikalen Lymphabflüsse erfasste den befallenen und die direkt angrenzenden Level (z. B. wurden bei einer LK-Metastase in Level II auch die Level I und V mit konturiert). Die ipsilateralen retropharyngealen LK wurden eingeschlossen bei LK-Metastasen in Level II oder einem Primarius mit Involvierung der posterioren pharyngealen Mukosa oder des posterioren Tonsillenbetts.

Die kontralateralen, nicht dissezierten Lymphabflüsse umfassten Level II, III und IV. Auf die Mitbehandlung dieser Lymphabflüsse konnte verzichtet werden bei einer invasiven Tumorbasis > 1 cm von der Mittellinie (intraoperative Einschätzung). Bei pN2c-Ausbreitung oder ipsilateralen retropharyngealen LK-Metastasen waren die kontralateralen Lymphabflüsse in jedem Falle mit zu behandeln.

## Ergebnisse

Insgesamt wurden zwischen 2013 und 2017 359 Patienten in die Studie eingeschlossen, davon 38 in Arm A, 100 in Arm B, 108 in Arm C und 113 in Arm D. Das mediane Alter war 58 Jahre, fast 90 % waren männlich, zu 66 % war der Primarius in der Tonsillenloge gelegen. Fast alle Tumoren zeigten ein pT1/2-Ausdehnung, fast die Hälfte einen pN2b-Lymphknotenbefall (multiple ipsilaterale LK, < 6 cm). Extranodales Wachstum wurde bei 37 % beschrieben, in ca. 3 % eine R1-Resektion.

Nach 35 Monaten Follow-up betrug das 2‑Jahres-PFS 97 % in Arm A, 95 % in Arm B (50 Gy), 96 % in Arm C (60 Gy) und fast 91 % in Arm D. Im Arm C wurden keine lokoregionären Rückfälle beobachtet im Vergleich zu 2 in Arm B, allerdings erlitten 4 vs. 2 Patienten Fernmetastasen. Das 2‑Jahres-OS betrug 100 % in Arm A, 99 % in Arm B (50 Gy), 98 % in Arm C und 96 % in Arm D.

Die Toxizität der adjuvanten Therapie unterschied sich jeweils signifikant zwischen den Behandlungsarmen und erreichte im Vergleich von Arm B, Arm C und Arm D für Grad 3 13 %/24 %/49 % und für Grad 4 2 %/0 %/11 %. Dabei stand erwartungsgemäß die orale Mukositis und Dysphagie im Vordergrund. Die Untersuchungen zur Lebensqualität (*Functional Assessment of Cancer Therapy-Head and Neck* [FACT-HN]) und zum Schlucken (*MD Anderson Dysphagia Index* [MDADI]) zeigten durch die Operation einen gleichmäßigen Abfall in allen Behandlungsarmen. Während sich die QoL der Patienten in Arm A dann zügig wieder erholte, fiel sie in den adjuvanten Behandlungsarmen zunächst weiter ab, um sich 6 Monate nach der Behandlung im Wesentlichen wieder normalisiert zu haben. Die MDADI-Werte hingegen blieben in den adjuvanten Therapiearmen auch 2 Jahre nach Abschluss der Therapie eingeschränkt, insbesondere im Vergleich zu Arm A.

## Schlussfolgerungen der Autoren

Bei HPV-induzierten Oropharynxkarzinom vom mittleren Risiko führen die primäre transorale Chirurgie und die reduzierte postoperative Radiotherapie (50 Gy, ohne simultane Chemotherapie) zu hohen Kurationsraten bei günstigen funktionellen Ergebnissen.

## Kommentar

Die hier besprochene Studie reiht sich ein in eine Serie von Untersuchungen zur Deeskalation der Therapie bei HPV-induzierten Oropharynxkarzinomen (Übersicht in [1]). Dabei ist sie die erste Arbeit, die prospektiv die Möglichkeiten der Therapiestratifizierung nach transoraler, minimal-invasiver Chirurgie nutzt und analysiert. In diesem Zusammenhang werden bei der ersten Zwischenanalyse hervorragende Daten in den Kollektiven mit niedrigem Risiko ohne weitere adjuvante Therapie berichtet und bei intermediärem Risiko; sowohl nach Standardradiotherapie als auch nach auf 50 Gy deeskalierter Radiotherapie. Dabei sind folgende Aspekte wichtig zu berücksichtigen:

1. Es handelt sich um einen ersten Zwischenbericht der ersten prospektiven Phase-II-Studie. Methodisch kann deshalb noch kein neuer Therapiestandard definiert werden. Aktuell rekrutiert die Phase-III-Studie *„Post-operative Adjuvant Treatment for HPV-positive Tumours*“ (PATHOS, NCT02215265) 1100 Patienten, erste Auswertungen sind erst in ca. 3 Jahren zu erwarten. Dennoch sind die Ergebnisse der E3311 gut und konkludent, sodass eine Therapie nach diesen Vorgaben den Patienten individuell nach entsprechender Aufklärung angeboten werden kann.
2. Die Definition der Risikogruppen basiert noch auf der 7. Auflage der TNM-Klassifikation, berücksichtigt also noch nicht den p16-Status. Mittlerweile werden für p16-positive Oropharynxkarzinome in der 8. Auflage bis zu 4 befallene Lymphknoten, unabhängig von der Größe der Metastasen als „pN1“ klassifiziert. D. h., bei einer Übertragung dieser Ergebnisse in die klinische Praxis muss unbedingt der pathologische Bericht detailliert betrachtet und gewürdigt werden, um eine korrekte Zuordnung in die jeweiligen Risikogruppen zu ermöglichen.
3. Weiterhin muss berücksichtigt werden, dass von den operativen Studienzentren eine ausführliche und strikte Akkreditierung zur Qualitätssicherung der operativen Ergebnisse und zur histopathologischen Aufarbeitung der Präparate verlangt worden war. Ob sich die so erzielten operativen Ergebnisse auch in der klinischen Routineversorgung reproduzieren lassen, wird die Zukunft zeigen.
4. Bezüglich einer Zuweisung der Patienten in Risikogruppen anhand postoperativer, histopathologisch erhobener Kriterien bietet eine solche zwar die Chance auf eine eindeutige, gut reproduzierbare Klassifikation. Dieser Konzeption inhärent ist allerdings auch das Risiko des „overtreatment“ der Hochrisikogruppe: Die „extensive ECE“ (also mindestens 1 mm) war bezeichnenderweise der häufigste Grund für die Zuweisung in den „Hochrisikoarm“ D. Diese Gruppendefinition setzt eine Operation zwingend voraus, da eine präoperative radiologische Abschätzung eines solchen Befunds derzeit nicht verlässlich möglich ist. Es ist aber fraglich, ob bei diesen Hochrisikopatienten ein „trimodales“ Vorgehen, bestehend aus Chirurgie und postoperativer RCT, onkologisch wirklich notwendig ist oder ob in diesen Fällen nicht a priori auf die Tumorresektion zugunsten einer alleinigen definitiven RCT verzichtet werden kann. Dieses legen zumindest die ORATOR-Daten nahe (allerdings nach robotischer Chirurgie und bisher bei einem nur kleinen rekrutierten Patientenkollektiv), die bei ähnlichen onkologischen Ergebnissen signifikant weniger Toxizität im MDADI-Gesamtscore nach RCT im Vergleich zur primären Operation (70 % zusätzlich adjuvante Therapie) zeigten [2].
5. Für die allein konservative Therapie der durch HPV induzierten Oropharynxkarzinome ist mittlerweile eindeutig belegt, dass der Standard eine cisplatinbasierte RCT ist und ein Ersatz von Cisplatin durch Cetuximab zu schlechteren onkologischen Ergebnissen führt [3, 4]. Große, prospektiv randomisierte Studien zur Deeskalation der Strahlendosis von 70 Gy in diesem Setting sind bislang noch nicht publiziert worden. In einem die kommentierte Studie im *JCO* begleitenden Editorial wird deshalb ein abgestuftes chirurgisches Vorgehen empfohlen, in dem primär eine Neck-Dissektion durchgeführt wird [5]. Bei Hochrisikokonstellation könnte dann die RCT ohne vorherige transorale Chirurgie appliziert werden. Ohne pathologische Risikofaktoren hingegen würden die Patienten zweizeitig die transorale Chirurgie und je nach Gesamtbefund eine entsprechend abgestufte (oder keine) adjuvante Therapie erhalten. Doch auch dieser „mehrzeitige chirurgische Ansatz“ kann kritisch hinterfragt werden, schließlich wäre die Neck-Dissektion bei der Mehrzahl der Patienten allein zur Risikostratifizierung notwendig. Nach primärer RCT ist sie onkologisch lediglich bei initial ausgedehntem Nodalbefall bei Tumorpersistenz auch 12 Wochen nach Therapieabschluss im PET-CT sinnvoll [6].

Zusammengefasst stellt die hier kommentierte Studie einen wichtigen Baustein zur Diskussion der Deintensivierung der Therapie bei ausgewählten Patienten nach transoraler Tumorresektion und Neck-Dissektion bei HPV-induzierten Oropharynxkarzinomen dar. Dennoch kann methodisch das Risiko einer Überbehandlung im Hochrisikokollektiv nicht aufgelöst werden (s. oben). Zudem wurde bei der adjuvanten Therapie in dieser Subgruppe keine Deeskalation versucht.

## Fazit

1. Patienten, die mit einem HPV-induzierten Oropharynxkarzinom transoral operiert wurden und ein „niedriges Risikoprofil“ aufweisen (pT1–2, R0 [mindestens 3 mm], pN0/N1 [1 LK-Metastase bis maximal 3 cm, ohne ECE], Pn0, L0), benötigen keine adjuvante Therapie.
2. Patienten, die mit einem HPV-induzierten Oropharynxkarzinom transoral operiert wurden und ein „mittleres Risikoprofil“ aufweisen (knappe Resektionsränder [< 3 mm], minimale ECE [< 1 mm], mindestens 1 LK-Metastase > 3 cm, 2–4 LK-Metastasen [< 6 cm] oder Pn1/L1), sind zumindest in dieser Studie mit 50 Gy adjuvanter Radiotherapie ohne begleitende Chemotherapie ausreichend behandelt. Da hier erstmals Daten von dieser einen Phase-II-Studie vorliegen, kann ein solches risikoadaptiertes Konzept noch nicht zum klinischen Standard erklärt werden, sollte aus unserer Sicht aber bei ausgewählten Patienten nach entsprechender Aufklärung bereits in der klinischen Routine angeboten werden.
3. Ob auch bei Patienten mit hohem Risikoprofil eine Therapiedeeskalation möglich ist, wird derzeit in anderen Studien untersucht.

*Robert Michael Hermann, Westerstede, und Hans Christiansen, Hannover*
